# Supplementary material for: MetaRibo-Seq measures translation in microbiomes
Source: Nat Commun. 2020 Jun 29;11:3268. doi: 10.1038/s41467-020-17081-z (PMC7324362; doi:10.1038/s41467-020-17081-z)
Supplement: Supplementary file 10 — Supplementary Data 7 [file 41467_2020_17081_MOESM10_ESM.zip › File2/Confidence_High_Taxonomy/70516_out.krona.html]

Javascript must be enabled to view this page.

members
magnitude
magnitudeUnassigned
count
unassigned
taxon
rank

70516\_out

29

superkingdom
29
1
2

SRS098065\_contig\_number\_15968

8
201174
phylum

class
1760
8

8
85009
order

family
8
31957

genus
8
1743

8
119981
species

SRS076868\_contig\_number\_contig-100\_10428.10429SRS077016\_contig\_number\_contig-100\_3721.42125SRS077633\_contig\_number\_contig-100\_1486.36479SRS104704\_contig\_number\_contig-100\_2401.42887SRS143529\_contig\_number\_21220SRS144560\_contig\_number\_46814SRS147712\_contig\_number\_contig-100\_7401.7402SRS148820\_contig\_number\_contig-100\_8541.79702

phylum
20
1239

186801
20
class


SRS147126\_contig\_number\_3079
20
1
186802
order

family
6
19
186803

SRS058336\_contig\_number\_19216SRS075404\_contig\_number\_14175SRS075765\_contig\_number\_9772SRS104165\_contig\_number\_contig-100\_1700.234660SRS104915\_contig\_number\_7939SRS149231\_contig\_number\_650


SRS077384\_contig\_number\_12107
species
1235800
1


SRS075987\_contig\_number\_17793SRS097895\_contig\_number\_31683SRS142787\_contig\_number\_29284SRS143604\_contig\_number\_34496
species
1951987
4

genus
1
1213720


SRS144644\_contig\_number\_7734
species
796942
1

189330
1
genus

88431
1

SRS146738\_contig\_number\_14751
species

species

SRS104314\_contig\_number\_21867
1
1898203

397287
3
species

SRS064774\_contig\_number\_9482SRS097912\_contig\_number\_12499SRS104087\_contig\_number\_20364

1408322
2

SRS097905\_contig\_number\_34031SRS098498\_contig\_number\_41975
species
